# The revision and factor analytic evaluation of the German version of the depression literacy scale (D-Lit-R German)

**DOI:** 10.1186/s40359-024-01730-9

**Published:** 2024-04-25

**Authors:** Feyza Gökce, Denise Jais, Philipp Sterner, Antonius Schneider, Jochen Gensichen, Gabriele Pitschel-Walz, For the POKAL-Group, Markus Bühner, Tobias Dreischulte, Peter Falkai, Jochen Gensichen, Peter Henningsen, Caroline Jung-Sievers, Helmut Krcmar, Kirsten Lochbühler, Karoline Lukaschek, Gabriele Pitschel-Walz, Barbara Prommegger, Andrea Schmitt, Antonius Schneider, Katharina Biersack, Constantin Brand, Vita Brisnik, Christopher Ebert, Julia Eder, Feyza Gökce, Carolin Haas, Lisa Hattenkofer, Lukas Kaupe, Jonas Raub, Philipp Reindl-Spanner, Hannah Schillok, Petra Schönweger, Clara Teusen, Marie Vogel, Victoria von Schrottenberg, Jochen Vukas, Puya Younesi

**Affiliations:** 1 School of Medicine and Health, Department of Clinical Medicine, Technical University Munich, Institute of General Practice and Health Services Research, Orleansstraße 47, 81667 Munich, Germany; 2 Department of Psychology, LMU Munich, Leopoldstr. 13, 80802 Munich, Germany; 3 Institute of General Practice, LMU Munich, Nußbaumstraße 5, 80336 Munich, Germany

**Keywords:** Depression literacy, Mental health literacy, Depression literacy scale, D-Lit-R German, Knowledge

## Abstract

**Background:**

Depression is a common mental health disorder and the second leading cause of disability worldwide. In people with depression, low depression literacy, which could be characterized by a poor recognition of depressive symptoms and less knowledge about the availability of treatment options, can hinder adequate therapy for depression. Nevertheless, questionnaires measuring depression literacy in Germany are rare. Consequently, for the present study, the German Depression Literacy Scale (D-Lit) has been revised and evaluated.

**Methods:**

First, a team of clinical psychologists revised the D-Lit German scale. Next, cognitive interviews were conducted with patients with depression to improve the comprehensibility of the scale items. Our revision of the D-Lit-R German scale was then subjected to an anonymous online study. Finally, the data went through an exploratory factor analysis, and sociodemographic subgroup analyses were performed.

**Results:**

*N* = 524 individuals (age 18–80) completed the D-Lit-R German scale and a questionnaire on their sociodemographic data. Cronbach´s alpha was estimated as α = .72, and McDonald's Omega (categorical) was estimated as ω = .77. The mean Item difficulty was* M* = .75 (*SD* = .15). An EFA was performed for a unidimensional model, a 5-factor-model and at last a 3-factor-model. The 5-factorial model showed a good model fit (χ^2^_emp,WLSMV_(131) = 92.424, *p* > .05; CFI = 1, RMSEA = 0, SRMR = .07) but was rejected since the content of the potential 5 factors could not be determined. The 3-factor model showed an arguable model fit. The Chi^2^ test was significant (χ^2^_emp,WLSMV_(168) = 199.912, *p* < .05), but the CFI and the RMSEA met an acceptable model fit (CFI = .990, RMSEA of .019, 90% CI[.003, .029]). Substantively, the three factors were defined as (1) Distractors and other symptoms, (2) Depressive symptoms, and (3) Pharmacological and psychotherapeutic depression treatment. Furthermore, there were significant differences in sum scores regarding the subgroup's gender, treatment for mental health problems, depression treatment, experience with depression, and different career fields.

**Conclusions:**

The D-Lit-R German scale is a time-efficient scale to assess some aspects of the depression literacy construct that can be easily applied. Since there was no perfect model fit, it is recommended to continue to revise the scale. Further evaluation studies could ask for knowledge of the etiological factors of depression. Future studies could then use this instrument to convey depression literacy. This instrument could assess the growth of knowledge after psychoeducational interventions in different settings.

**Trial registration:**

This trial was preregistered at the platform osf.io (https://osf.io/49xdh).

Registration number: https://doi.org/10.17605/OSF.IO/49XDH

Date of registration: 28 April 2022.

**Supplementary Information:**

The online version contains supplementary material available at 10.1186/s40359-024-01730-9.

## Background

With a prevalence rate of 28.9%, depression is now a worldwide mental disorder [1]. This disorder is characterized by the main symptoms of a depressed mood, decreased energy, and lack of interest for at least two weeks. Several other, partly somatic symptoms include sleep disturbances and loss of appetite [2]. In Germany, even though effective treatments for depression already exist and are commonly used, e.g., cognitive behavioral psychotherapy, interpersonal psychotherapy, or antidepressant medication [3], more than half of the people with depression don´t use the available health services [4]. In addition to access barriers, e.g., inability to get an appointment or long waiting times, individual barriers also play an essential role in explaining this [5]. One barrier can be the fear of being stigmatized. Another barrier can be dysfunctional cognitions, e.g., believing that treatment will not be helpful or wanting to deal with the problem on their own. Tomczyk et al. (2018) found that informal help-seeking was negatively associated with depression literacy, meaning that people with high depression literacy were less likely to seek informal help and vice versa [6]. In the literature, depression literacy or depression knowledge is defined as a sub-construct of mental health literacy (MHL) [7]. Depression literacy includes knowledge about depressive symptoms, depression-relevant support services, and competence in applying this knowledge. MHL, in general, refers to the knowledge of how to identify a specific mental disorder, how to access health information, what the risk and etiological factors are, and what self-help and treatment options are available [8]. MHL also comprises attitudes that promote appropriate treatment. Greater MHL is shown to predict the readiness to take up treatment [9, 10]. In Contrast, lower depression literacy is associated with poor recognition of depressive symptoms and less knowledge of the availability of treatment options [11]. Sociodemographic factors such as age, gender, and general education can also influence MHL. Studies show that MHL is lower in older adults, in people with lower education, and in men compared to women [12–14].

Regarding depression literacy, Makowski et al. (2021) conducted a national telephone study and found that 55% of the participants recognized depression as the health problem depicted in a case vignette [15]. Although this seems to be a moderate level of depression literacy, it is of great importance to increase depression literacy in society, in view of the growing number of people with depression worldwide [16]. More services should be offered to particularly vulnerable groups to improve the course of depressive disorders and reduce barriers to access. This could be achieved, for example, through a psychoeducational intervention in General practices, as most patients with depression first consult their GP and are also treated by them [17–19]. Instruments used to assess MHL are mainly case vignettes that ask for disease-specific declarative knowledge [20]. Those vignettes focus on the knowledge of terminology, risk factors, diagnosis, and prognosis, which are captured by recognition tests [8]. In addition, there are also several standardized knowledge questionnaires on MHL [20]. Wei et al. (2018) identified a total of 69 knowledge questionnaires in a review of available MHL measurement instruments from 401 studies, most notably the Mental Health Literacy Scale (MHLS) [21, 22]. However, questionnaires measuring specific disease-related knowledge about depression in Germany are insufficient. One example is a knowledge questionnaire by Görnitz et al. (1998), which has not yet been evaluated [23]. Since there is a lack of evaluated questionnaires in the field of depression knowledge, Freitag et al. (2018) translated the depression literacy scale (D-Lit) by Griffiths et al. (2004) [24] into German and conducted an evaluation study [7]. The original scale consists of 22 items, and the translated German scale had the same properties as the original scale and reached an internal consistency of Cronbach's alpha = 0.747, which can be regarded as satisfactory [25]. However, the authors state that there may have been problems with the content or language of some items in terms of comprehension.

Furthermore, it should be noted that the sample used in the study consisted of individuals with depressive symptoms. Therefore, the authors recommend evaluating the scale on a subclinical sample. Consequently, the present study aimed to optimize the D-Lit German scale as a measure of depression knowledge and to test its psychometric properties and factor structure. Furthermore, it aimed to investigate the differences in depression literacy regarding subgroups based on psychosocial variables (e.g., Gender, Age) to select those with a greater need for interventions that increase depression literacy. Overall, this study was conducted to develop a validated instrument that can be used in another subsequent study to assess depression knowledge in patients with depression. In this planned study, a psychoeducation program for patients with depression [26] will be tested in general practices. Since one of the variables of interest will be depression literacy, it was also aimed to optimize the compatibility of the D-Lit German scale as a measuring instrument for such an intervention.

## Methods

### Study design

This study was preregistered at the platform osf.io (Registration link: https://osf.io/49xdh, registration number: https://doi.org/10.17605/OSF.IO/49XDH). The study consisted of two parts. The first part included the revision of the scale. Part two comprised the online survey to investigate psychometric properties, analyze the scale's factor structure, and compare differences in the sum score depending on sociodemographic subgroups. Before data collection, the study was assessed and approved by the ethics committee of the Technical University of Munich (TUM). All methods were carried out in accordance with relevant guidelines and regulations. All participants confirmed their informed consent before taking part in the study.

### Adaptation and revision

First, the existing D-Lit German scale [7] was revised by an expert team of two clinical psychologists to improve the comprehensibility of the scale items as recommended by Freitag et al. (2018) [7]. Four items were found to be misleading or not in accordance with the current S3 guidelines for depression. These items were replaced by four new items, thematizing other aspects of depression and depression treatment. This first draft of the revised D-Lit-R German scale [27] was developed for the present study, has not been published before, and is available in the online supplements. The scale then underwent a pretest with cognitive interviews with the technique of “thinking aloud” [28] to identify possible comprehension and response problems. In accordance with the recommendation of Prüfer & Rexroth (2005) [28], we aimed to recruit at least 5 participants for the interviews. Due to difficulties in the recruitment, the final number of participants was *N* = 5, consisting of patients with depression (two women and three men; Ages: 24, 28, 43, 49, and 66) staying at the private ward of the Clinic for Psychiatry and Psychotherapy of the University Hospital Rechts der Isar of the Technical University in Munich (TUM). The Interviews were conducted by one of the first authors of the present study (DJ) in April 2022. They were audio recorded and transcribed. After transcription, the audio records were deleted. Participants gave informed consent before taking part in the interviews.

The registered problems in the transcripts were rated following the Question Rating System of Faulbaum et al. (2009) [29]. Then, the altered items were again discussed and reviewed by the clinical psychologists and the study team.

### Online survey

In a subsequent online survey, a convenient sample of participants answered the modified D-Lit-R German scale [27] and a sociodemographic questionnaire. All interested individuals (18–80 years) who did not meet exclusion criteria were eligible to participate and thus included. The exclusion criteria comprised cognitive impairment, language barriers, an academic degree in psychology, or another expertise in psychology or psychiatry. Interested persons were only invited to participate in the study if they did not meet any exclusion criteria. Expertise in psychology or psychiatry was further assessed by asking questions about their profession in the sociodemographic questionnaire at the beginning of the online survey. The survey was conducted via the internet platform www.sosci-survey.de. The period for participation was 6 weeks (May–June 2022). The time required to complete the survey was suggested to be 5–10 min. Subjects were asked to give their informed consent at the beginning of the survey by clicking a button since data collection was anonymous. Participation was voluntary and unpaid. By closing the survey web page, participants could end the survey at any time if they no longer wished to participate.

### Sample size and recruitment

Schönbrodt and Perugini (2013) recommend a minimum sample size of *N* = 250 since stable estimates of correlations with manifest variables can only be expected with this sample size [30]. For stable estimates regarding the correlations of latent variables, a sample size of 490 persons is recommended [31]. Therefore, a target sample size of *N* = 490 individuals was aimed for.

Recruitment was conducted via analog and digital advertisement through flyers at universities, clinics, other educational institutions, and institutions of daily life (e.g., supermarkets) in Munich, Germany, and Innsbruck, Austria.

### Measurement tools

#### Sociodemographic questionnaire

The questionnaire included items with questions on the following domains: Age, gender, experience with treatment for mental health problems, experience with depression (self, acquaintance), current treatment for depression, level of education, and career field. We further assessed how the participants found out about the study.

#### D-Lit-R German scale [27]

A revised version of the German translation of the Depression Literacy Scale (D-Lit German) by Freitag et al. (2018) [7] was used to assess depression literacy. The original version was constructed by Griffiths et al. (2004) [24]. In total, the revised D-Lit-R German scale [27] that we developed for this study contains 22 items with a three-part response format ("true," "false," "I don't know"). One point is given for each correct answer; no point is awarded for questions answered incorrectly or with "I don't know.". A higher sum score indicates a greater depression knowledge [7]. Freitag et al. (2018) [7] report a Cronbach’s α of 0.747 for the D-Lit German scale. Four items of the D-Lit German scale were replaced by reformulated items (see Table 1). However, the revised scale still had the same response format as the D-Lit German scale and was evaluated and interpreted in the same way. The authors of both the original and the translated version of the D-Lit scale, Kathleen Griffiths and Simone Freitag, have kindly given their permission to use their versions for the further development of the scale.

**Table 1:** Modified/ Replaced Items and their original version

Item	Original Item	Modification by expert team	Modification after cognitive interviews
(1)	People with depression often speak in a digressive and incoherent way		People with depression often speak incoherently
(3)	Reckless and foolhardy behaviour are common signs of depression		Reckless and risk-taking behaviour are common signs of depression
(12)	Clinical psychologists can prescribe antidepressants		Psychologists can prescribe antidepressants
(13)	Moderate depression disrupts a person's life as much as multiple sclerosis or deafness	Depression can be accompanied by distortions in thinking and perception	Depression can be accompanied by changes in thinking and perception (e.g. brooding)
(15)	Many famous people have suffered from depression		Depression always has several causes
(16)	Many treatments for depression are more effective than antidepressants	Increasing positive activities should be an integral part of any treatment for depression	Increasing positive activities (e.g. exercise, socializing) should be an integral part of any treatment for depression
(18)	For mild to moderate depression, cognitive behavioural therapy is as ef- fective as antidepressants	For mild to moderate depression, cognitive behavioural therapy is the treatment of choice	
(19)	Of all the alternative and lifestyle treatments for depression, vitamins are probably the most helpful	People with depression have negative thoughts, which can also take the form of suicidal thoughts	

### Statistical analysis

Statistical analyses were performed using SPSS statistical software version 27 (IBM SPSS Statistics) and R (version 4.2.0 R Core Team, 2022) and the packages Jmv (Version 2.3.4;), Lavaan (Version 0.6–12; Rosseel, 2012), MBESS (Version 4.9.1; Kelley, 2017), nfactors (Version 2.4.1; Raiche, 2010), psy (Version 1.2; Falissard & Falissard, 2022), psych (Version 2.2.5; Revelle, 2022), and RE- daS (Version 0.9.4; Maier, 2022). The significance level was set at α = 0.05 for two-sided significance tests. For multiple tests, the significance level was corrected according to Bonferroni. Multiple imputations (chained equation) [32] of missing values of the dependent variable (D-Lit-R German data) were not performed since the small number of three data points (0.03%) did not follow a systematic pattern and were classified as MCAR values (missing completely at random) [33, 34].

Descriptive item statistics (means, standard deviations, selectivity, item skewness) were calculated, and distributional analyses (scale skewness and scale kurtosis) were performed. The scale's reliability was assessed using Cronbach´s alpha and McDonald's Omega (categorical). To check the suitability of the present data for the factor analysis, we examined Bartlett's test for sphericity [35] with the Kaiser–Meyer–Olkin coefficient (KMO) [36] and the correlations of the anti-image matrices (MSA coefficients). This was followed by a factor analytic examination of the D-Lit-R German scale with determination of the number of factors to be extracted using parallel analysis [37] and MAP test [21, 38]. Because of the categorical response format and the fact that the factors would correlate, a WLSMV (Weighted Least Squares Mean Variance – adjusted; rotation: oblimin) factor analysis was calculated [39, 40]. Measurement models were evaluated using the following fit indices: global model fit (Chi2 test), RMSEA (Root Mean Square Error of Approximation), CFI (Comparative Fit Index), and SRMR (Standardized Root Mean Square Residual) [41]. We want to point out that we used these fit indices to compare models with varying numbers of factors. In this, we tried to find a trade-off between model fit and model interpretability. Finally, subgroup analyses were performed using t-tests (Welch tests for missing prerequisites), nonparametric tests (Mann–Whitney U test), and the univariate analyses of variance complemented by Dunnett T3 post hoc tests.

## Results

### Adaptation and revision

In sum, 10 original D-Lit German scale items were replaced by reformulated items and modified. Table 1 shows the modified items and their original versions. There were several reasons for the modification or replacement of the items. Items 1, 3, and 12 were slightly modified to improve the comprehensibility. Item 18, which initially had to be answered with “correct”, is a question on the treatment of depression that is not in line with the current recommendations of the S3- guidelines. Therefore, we replaced it with a more accurate item regarding depression treatment based on the guidelines' recommendations. Items 13, 16, and 19 were found to be misleading and display implausible comparisons. These items were replaced by new items, which were chosen in accordance with the content of the psychoeducational program of the subsequent study, for which the revised scale is thought to be one of the measuring tools. The same reasoning was crucial for the replacement of item 15. The original item 15 was found to be too easy based on the feedback in the cognitive interviews. Since the original item possibly aimed to decrease stigma, we chose to replace it with an item about the causes of depression that could also have a destigmatizing effect.

### Descriptive statistics

630 subjects participated in the study. Before data analysis, 103 data entries were excluded since only the link had been clicked on and no data had been entered. Additionally, 3 subjects were excluded from the analysis because they met an exclusion criterion. Table 2 shows the descriptive statistics of the sociodemographic characteristics of the final sample (*N* = 524). Most participants stated that they became aware of the study through their university or college (53.2%), and some have been approached by friends, family, or acquaintances (28.6%). Other participants were asked to participate through the Institute of General Practice and Health Services Research (10.9%) and some through social media (6.9%). Regarding the gender of the participants, the sample included 370 (70.6%) women and 142 (27.1%) men, 7 (1.3%) participants choosing the option “diverse” and 5 (1%) participants selecting the response option "I do not wish to provide information". The sample's age ranged from 18 to 79 years (M = 32.38; SD = 14.69).

**Table 2:** Sociodemographic characteristics of the final sample (*N* = 524)

Variable	Variable Category	*n* (%)
Age	*Mean* (*SD*)	32.38 (14.69)
Gender	Female	370 (70.6)
	Male	142 (27.1)
	Divers	7 (1.3)
	No information	5 (1.0)
Experience with Treatment for mental disorders	yes	228 (43.5)
	no	288 (55.0)
	No information	8 (1.5)
Experience with Depression (multiple answers possible)	Yes, myself	203 (38.7)
	Yes, as an acquaintance	164 (31.3)
	Yes, other	121 (23.1)
	No	112 (21.4)
Current Depression Treatment	Psychotherapy	80 (15.3)
	Psychiatric Treatment	48 (9.2)
	Other Treatment	14 (2.7)
	No Treatment	412 (78.6)
	No information	5 (1.0)
Level of Education (School diploma)	No school	2 (0.4)
	Special education school	2 (0.4)
	Lower secondary school	11 (2.1)
	Secondary school	56 (10.7)
	Abitur	449 (85.7)
	Missing	4 (0.8)
Career field	Technology	43 (8.2)
	Social Field	112 (21.4)
	Economy	68 (13.0)
	Health Sciences	70 (13.4)
	Humanities	65 (12.4)
	Nature Sciences	78 (14.9)
	Legal Studies	33 (6.3)
	Other career field	50 (9.5)
	No information	5 (1.0)

*n* = number of subjects choosing this category, *SD *Standard Deviation

### Online survey

Data from *N* = 524 subjects could be included in the statistical analysis; the mean sum score was *M* = 16.52 (*SD* = 3.40), the selectivity of the items ranged between *r*_*it*_ = -0.02—0.50, with 15 of the 22 items (68.2%) having a selectivity < 0.30, which can be considered as a moderate degree [39, 42]. Since Cronbach´s alpha was α = 0.72 and McDonald's Omega (categorical) was ω = 0.77, the scale's reliability was found to be acceptable. Table 3 shows the descriptive statistics.

**Table 3:** Descriptive statistics of the German D-Lit-R scale

Item		*M*	*SD*	*r*_*it*_	*ω*	*V*
(1)	People with depression often speak incoherently	.58	.49	.23	.73	-.34
(2)	People with depression can feel guilty even though they have done nothing wrong	.96	.20	.16	.73	-4.70
(3)	Reckless and risk-taking behaviour are common signs of depression	.68	.47	.25	.73	-.76
(4)	Loss of self-confidence and low self-esteem can be signs of depression	.98	.14	.08	.74	-6.70
(5)	Not stepping on the joints of a footpath can be a sign of depression	.68	.47	.35	.72	-.76
(6)	People with depression often hear voices that are not there	.75	.43	.48	.71	-1.18
(7)	Sleeping too much or too little can be a sign of depression	.90	.29	.29	.72	-2.76
(8)	Eating too much or losing your appetite can be signs of depression	.92	.28	.28	.72	-3.01
(9)	Depression does not affect memory and concentration	.84	.37	.25	.73	-1.85
(10)	Having several different personalities can be a sign of depression	.64	.48	.50	.71	-.58
(11)	As a result of depression, people may move more slowly or be completely restless	.84	.36	.17	.73	-1.9
(12)	Psychologists can prescribe antidepressants	.68	.47	.42	.72	-.77
(13)	Depression can be accompanied by changes in thinking and perception (e.g. brooding)	.95	.21	.21	.73	-4.36
(14)	Most people with depression need to be admitted to hospital	.95	.21	.29	.72	-4.36
(15)	Depression always has several causes	.59	.49	-.02	.75	-.36
(16)	Increasing positive activities (e.g. exercise, socializing) should be an integral part of any depression treatment	.90	.30	.17	.73	-2.72
(17)	For depression, counselling is as effective as cognitive behavioural therapy	.29	.45	.28	.73	.95
(18)	For mild to moderate depression, cognitive behavioural therapy is the treatment of choice	.47	.50	.23	.73	.22
(19)	People with depression have negative thoughts, which can also take the form of suicidal thoughts	.98	.14	.21	.73	-7.05
(20)	People with depression should stop taking antidepressants as soon as they feel better	.76	.43	.39	.72	-1.22
(21)	Antidepressants are addictive	.51	.50	.46	.71	-.02
(22)	Antidepressants usually work immediately	.72	.45	.44	.72	-.99

*M *Mean, *SD *Standard Deviation, *r*_*it*_Selectivity, *ω *McDonalds´s Omega when Item is removed, *V *Skewness of Item Skewness of scale = -.93, Kurtosis = 1.75. Item difficulty varied between .29—.98 (*M* = .75; *SD* = .15)

The mean completion time of the online survey was *M* = 4.36 (*SD* = 1.95) minutes. Since participants could have looked up some of the answers online, a spearman correlation analysis of the completion time and the sum score was conducted. A significant, negative correlation of *r* = -0.153, *p* < 0.001 showed that a longer completion time was associated with a smaller sum score.

### Factor analysis

Based on the Kaiser–Meyer–Olkin coefficient (KMO = 0.77), the data was considered suitable for the factor analysis. In addition, the anti-image matrices of the inter-item correlations had high MSA coefficients (0.51—0.86). The Bartlett's test of sphericity showed that all correlations were significantly different from zero (χ^2^ = 1584.35; *p* < 0.001; df = 231). Horn's (1965) parallel analysis suggested extracting 5 factors for which eigenvalues are reported above the 95% percentile [37]. However, because the MAP test extracted only one factor for the minimum mean squared partial correlation (0.01) and since it was assumed that the scale measured the construct depression knowledge, an EFA (algorithm: WLSMV, rotation: oblimin) was first performed for a unidimensional model.

### Unidimensional model

Table 4 shows the factor loadings (λ) and commonalities (*h*^*2*^). The unidimensional structure accounted for 28.5% of the total variance.

**Table 4:** Item parameters for a unidimensional model

**Item**		***λ***	***h***^***2***^
(1)	People with depression often speak incoherently	.35^**^	.12
(2)	People with depression can feel guilty even though they have done nothing wrong	.42^*^	.18
(3)	Reckless and risk-taking behaviour are common signs of depression	.34^**^	.12
(4)	Loss of self-confidence and low self-esteem can be signs of depression	.26	.07
(5)	Not stepping on the joints of a footpath can be a sign of depression	.57^**^	.32
(6)	People with depression often hear voices that are not there	.81^**^	.66
(7)	Sleeping too much or too little can be a sign of depression	.61^**^	.37
(8)	Eating too much or losing your appetite can be signs of depression	.62^**^	.38
(9)	Depression does not affect memory and concentration	.42^**^	.18
(10)	Having several different personalities can be a sign of depression	.81^**^	.66
(11)	As a result of depression, people may move more slowly or be completely restless	.26^**^	.07
(12)	Psychologists can prescribe antidepressants	.64^**^	.41
(13)	Depression can be accompanied by changes in thinking and perception (e.g. brooding)	.44^**^	.19
(14)	Most people with depression need to be admitted to hospital	.69^**^	.48
(15)	Depression always has several causes	-.05	.00
(16)	Increasing positive activities (e.g. exercise, socializing) should be an integral part of any depression treatment	.25^*^	.07
(17)	For depression, counselling is as effective as cognitive behavioural therapy	.46^**^	.21
(18)	For mild to moderate depression, cognitive behavioural therapy is the treatment of choice	.28^**^	.08
(19)	People with depression have negative thoughts, which can also take the form of suicidal thoughts	.58^**^	.34
(20)	People with depression should stop taking antidepressants as soon as they feel better	.64^**^	.41
(21)	Antidepressants are addictive	.73^**^	.53
(22)	Antidepressants usually work immediately	.65^**^	.42

^**^p < .001

^*^p < .05

*λ* = loadings

*h*^*2*^ = commonalities

A total of 19 loadings were significant at a significance level of *p* < 0.001 and two loadings (*λ*_*2*_*, λ*_*16*_) at a significance level of *p* < 0.05. The chi^2^ test calculated for the general model fit was significant (χ^2^_emp,WLSMV_(35) = 173.252, *p* < 0.001), the model is therefore rejected. Similarly, the fit indices of the single-factor model (CFI = 0.930, RMSEA = 0.087, SRMR = 0.146) failed to meet the cut-off values defined by Hu and Bentler (1999) [41]. Due to the rather poor model fit, the single-factor- congeneric model was rejected based on the fit indices and the chi2 test.

### 5-factor-model

Based on the parallel analysis, an EFA was calculated for a multidimensional τ-congeneric model. The 5-factorial structure explained 52.61% of the total variance. The chi^2^ test was not significant (χ^2^_emp,WLSMV_(131) = 92.424, *p* > 0.05). The fit indices also indicated a good model fit (CFI = 1, RMSEA = 0, SRMR = 0.07). Although the 5-factorial model seemed to explain the data statistically best, the content of the factors could not be sufficiently depicted, respectively.

### 3-factor-model

As a result, the model was discarded in favor of a 3-factor model based on theoretical post-hoc considerations regarding the content of the factors. The explained total variance of the 3-factorial model was 42.62%. The proportion of correct answers varied between the factors. The global hypothesis test was significant, χ^2^_emp,WLSMV_(168) = 199.912, *p* < 0.05. The CFI is 0.990, arguing for acceptance of the model, followed in the output, by the RMSEA of 0.019, 90% CI[0.003, 0.029], which meets an acceptable model fit. The SRMR of 0.093 failed to meet the common cut-offs by Hu and Bentler (1999) [41]. Six items had higher loadings on factor 1, nine items on factor 2, and six on factor 3 (Table 5). Item 15 had the lowest loading (λ_15_ = 0.04), which did not become significant on any of the factors. This item did not seem to belong to any factor, so it should be removed from the scale in the long term. However, the remaining 21 loadings were significant on at least one factor. Other modified items (1, 3, 12, 13, 16, 18, 19) had factor loadings and communalities of λ = 0.18—0.75 and *h*^2^ = 0.10—0.71. The three highest loadings were on item 8 (λ_8_ = 0.89), item 10 (λ_10_ = 0.85) and item 22 (λ_22_ = 0.80). Item 3 had a double loading on the first (λ_3_ = 0.18) and second factors (λ_3_ = 0.19).

**Table 5:** Factor loadings on a 3-factor model

			Factors		
Item		*h*^*2*^	1	2	3
(1)	People with depression often speak incoherently	.13	.21^*^	.12	.15
(3)	Reckless and risk-taking behaviour are common signs of depression	.13	.18^*^	.19^*^	.11
(5)	Not stepping on the joints of a footpath can be a sign of depression	.55	.79^**^		
(6)	People with depression often hear voices that are not there	.74	.76^**^	.11	.12
(10)	Having several different personalities can be a sign of depression	.83	.85^**^		.13^*^
(14)	Most people with depression need to be admitted to hospital	.55	.54^**^	.29^*^	
(2)	People with depression can feel guilty even though they have done nothing wrong	.30		.50^*^	
(3)	Reckless and risk-taking behaviour are common signs of depression	.13	.18^*^	.19^*^	.11
(4)	Loss of self-confidence and low self-esteem can be signs of depression	.35		.63^**^	
(7)	Sleeping too much or too little can be a sign of depression	.60		.72^**^	.14
(8)	Eating too much or losing your appetite can be signs of depression	.80		.89^**^	
(9)	Depression does not affect memory and concentration	.27		.43^**^	.18
(11)	As a result of depression, people may move more slowly or be completely restless	.23		.45^**^	.21^*^
(13)	Depression can be accompanied by changes in thinking and perception (e.g. brooding)	.32	.18	.48^**^	
(16)	Increasing positive activities (e.g. exercise, socializing) should be an integral part of any depression treatment	.10	.14	.26^*^	
(19)	People with depression have negative thoughts, which can also take the form of suicidal thoughts	.71	.29^*^	.75^**^	
(12)	Psychologists can prescribe antidepressants	.44	.23^**^	.26^**^	.39^**^
(17)	For depression, counselling is as effective as cognitive behavioural therapy	.25	.18		.39^**^
(18)	For mild to moderate depression, cognitive behavioural therapy is the treatment of choice	.10		.10	.25^*^
(20)	People with depression should stop taking antidepressants as soon as they feel better	.63	.10		.76^**^
(21)	Antidepressants are addictive	.70	.20^**^		.74^**^
(22)	Antidepressants usually work immediately	.66		.15^*^	.80^**^
(15)	Depression always has several causes	.01			

^**^p < .001

^*^p < .05

λ loadings

*h*^*2*^ commonalities. For clarity, factor loading < .10 are left blank

### 3-factor-model after removing Item 15

After removing Item 15, which did not have a significant loading on any factor, the analysis was conducted again for the 3-factor model, resulting in a better model fit with *χ*^*2*^_*emp,WLSMV*_(150) = 156.062, *p* > 0.05, CFI = 0.998, RMSEA = 0.009, SRMR = 0.088. The explained total variance of the 3-factor model without item 15 increased to 44.78% and the items still loaded on the same factors as before. The individual factor loadings can be found in the online supplements.

### Content identification of the potential factors

#### Factor 1: distractors and other symptoms

Factor 1 is composed of items that are predominantly not associated with the construct depression and mainly address symptoms of other mental illnesses. Items 1, 3, 5, 6, 10 and 14 therefore discriminate incorrect from correct knowledge about depressive symptoms.

#### Factor 2: depressive symptoms

This factor represents items that primarily ask about depressive symptoms (2, 4, 7, 8, 9, 11, 13, 16, 19). Item 16 had the lowest communality and more likely corresponds to the treatment of depression.

#### Factor 3: pharmacological and psychotherapeutic depression treatment

This factor contains items regarding the pharmacological or psychotherapeutic treatment of depression (12, 17, 18, 20, 21, 22). Item 15 did not seem to be connected to any factor.

#### Sociodemographic subgroup analyses

There was no significant influence of age on the number of correct answers. Due to the very small number of cases in the gender groups ‘divers’ and ‘no information’, only the gender groups of women and men were compared. There were statistically significant differences between the two gender categories (t_Welch_(237,45) = 4.37, *p* < 0.001, d = 0.45), with females averagely scoring 1.5 more correct responses than males. Differences in the sum score depending on the level of education were analyzed by a t-test for the two most predominant groups of education, Abitur and secondary school. The results of the Welch test showed no statistically significant differences between the groups. Using the Mann–Whitney U-test, a significantly higher sum score was found for people who had already sought treatment for mental health problems compared to people without treatment experience (U = 21,494, Z = -6.771, *p* < 0.001, *r* = 0.30). Also, using the Mann–Whitney U-test, a significant difference was found between the group that had been in depression treatment before and the group without previous depression treatment, with higher scores in the first group (U = 14,494, Z = -5.71, *p* < 0.001, *r* = 0.25). Additionally, another Welch test revealed statistically significant differences between the group that had already been affected by depression themselves or had someone close to them who was affected and the group without any experience depression (t_Welch_(175,81) = 6.54, *p* < 0.001, *d* = 0.74). Individuals who had experience of depression, scored on average 2.4 more correct responses than those without experience. Significant mean differences were also found between the different career fields (technical, social, economic, health sciences, humanities, natural sciences, law, other field of activity, not specified) using a one-factor ANOVA with post-hoc tests (Dunnett-T3) (F(8, 515) = 132.28, *p* < 0.001, η^2^ = 0.174, 95% CI[0.108, 0.221] and are depicted in Table 6.

**Table 6:** Differences between the sum scores in Dunnet-T3 Post-hoc Test regarding the career field of subjects

	M_diff_ (SE), *p*-Value							
CF	T	SF	E	HE	HU	NS	LS	Other
T		-2.06^*^ (.57), *p* = .012	.20 (.71), *p* > .999	-3.26^**^ (.57), *P* < .001	-1.69 (.58), *p* = .124	-1.57 (.57), *p* = .174	-1.30 (.61), *p* = .623	-.45 (.67), *p* > .999
SF			2.26^**^ (.60), *p* = .008	-1.20 (.42), *p* = .122	0.36 (.44), *p* > .999	0.49 (.42), *p* > .999	0.76 (.48), *p* = .58	1.60 (.55), *p* = .118
E				-3.46^**^ (.61) *P* < .001	-1.89 (.63) *p* = .084	-1.77 (.61) *p* = .120	-1.50 (.66) *p* = .476	-0.66 (.71) *p* > .999
HE					1.57^*^ (.46) *p* = .022	1.69^**^ (.44) *p* = .004	1.96^**^ (.49) *p* = .004	2.80^**^ (.56) *p* < .001
HU						0.12 (.46) *p* > .999	0.39 (.51) *p* > .999	1.24 (.58) *p* = .607
NS							0.27 (.49) *p* > .999	1.12 (.56) *p* = .736
LS								0.845 (.61) *p* = .990

M_diff _Mean Difference,  *SE* Standard Error, *p *Significance Value, *CF *Career field, *T *Technology, *SF *Social Field, *E *Economy, *HE *Health sciences, *HU *Humanities, *NS *Nature sciences, *LS *Legal studies

^*^p < .05

^**^ *p* < .01

## Discussion

In the present study, we revised and examined the German Depression Literacy Scale [7] regarding its factoranalytic and psychometric values. As recommended by Freitag et al. (2018) [7], we conducted the revised German Depression Literacy Scale [27] in a convenient sample.

The parallel analysis within the EFA revealed a 5-factorial structure of the D-Lit-R German scale, contrary to the assumption that depression knowledge could be a unidimensional construct as it had been extracted by the MAP test. After examining the unidimensional model and reviewing the fit indices, the assumption of a single-factorial structure was rejected. Although the 5-factor model fitted the data better than the unifactorial model, we could not make sense of the content of a 5-factor model. We chose the 3-factor model because it is a good trade-off between interpretability and model fit. Freitag et al. (2018) postulated that the original German version contained items regarding depressive symptoms and knowledge of other psychological symptoms [7]. These two categories were also reflected by the results of the present study. Jorm (2012) defines mental health literacy as a composition of many factors:1. knowing how to prevent mental disorders, 2. recognizing when a disorder is developing, 3. knowing what help-seeking options and treatments are available, 4. knowing which effective self-help strategies for milder problems exist, and 5. having first aid skills to support others who are developing a mental disorder or are in a mental health crisis [43]. Two of these domains could be identified in the 3-factorial model in the present study. One factor (factor 2) contained items testing knowledge about depression symptoms. The other factor (factor 1) also entailed items that tested symptom knowledge but related to symptoms of other mental disorders. As mentioned above, the third factor asked for items related to treatment knowledge. Treatment knowledge (factor 3) and symptom knowledge (factors 1 and 2) are only two of the essential components of depression knowledge [7, 43]. The other constructs subsumed under depression knowledge, such as prevention knowledge, help-seeking knowledge, and informal support options [43], were not represented in the modified D- Lit-R German scale.

These findings complement the literature on studies that translated and validated the original D-Lit scale into other languages. The factors extracted by the factor analysis we conducted are similar to the results of Jeong et al. (2017) [44], which also retained 3 factors for their revised 21-item Korean version of the D-Lit scale: 1) misperceptions about depression and its treatment; 2) knowledge about depression; and 3) knowledge about the treatment of depression. Other studies have detected either a 5-factor model [45] with factor domains similar to what we suggested or a 1-factor model, covering depression literacy as a one-dimensional construct [46, 47].

In the present study, we aimed to adapt and revise the scale to increase the comprehensibility and correct items that were not aligned with the S3 guidelines. Furthermore, we tried to match the items of the scale to the topics that will be presented in a psychoeducation program in a subsequent study, which concerns improving depression care in general practices. Since most translations had been adapted for cultural aspects [44–46, 48], the different versions might, in fact, represent different domains of depression literacy. To create a scale that represents all of the domains of depression literacy based on the MHL definition by Jorm (2012), further studies should adapt the D-Lit scale according to his definition.

For our final version of the D-Lit-R German scale, we recommend excluding Item 15 since it shows a low selectivity, and excluding it increased the explained total variance. Compared to the reliability of the original German version of the D-Lit-German scale (α = 0.75) [7], the reliability of our scale in the present study (α = 0.72; ω = 0.77) was similar. The reliability analysis of the English version revealed a Cronbach's alpha of 0.70 with a test–retest reliability of 0.71 [24] and is slightly below the reliability of the D- Lit-R German scale [27]. A majority of almost 75% of the respondents answered the questions correctly, which is notably higher than the 50% correct response rate detected by Freitag et al. (2018) [7]. Respectively, the percentage of correct answers for symptom knowledge, which excludes incorrect symptoms (factor 1) and identifies correct symptoms (factor 2), was 71% and 91%. This means that the sample has a high level of symptom knowledge. Compared to that, only 38% correctly answered questions regarding the possible third factor, which is depression treatment (pharmacological and psychotherapeutic treatment). This supports findings from previous studies on depression knowledge [7, 49, 50] and implies that the present sample seems to have less knowledge regarding guideline-based treatment.

To our knowledge, the present study is the first attempt to analyze the factor structure of the German D-Lit scale. Having an instrument to measure depression literacy without using case vignettes is important to enable standardized research since most studies use different kinds of case vignettes, which often leads to results that are difficult to compare [51, 52]. Although it was possible for researchers like Makowski et al. (2021) [15] to gain representative results on depression literacy by using case vignettes, this approach requires many resources. By revising the German D-Lit scale, we promote the extensive usage of this short and time-saving scale in German mental health research. Furthermore, we replaced misleading items with items that matched to a cognitive-behavioral depression psychoeducation [26]. Therefore, it could be expected that the scale can also be useful to measure the growth in depression literacy after other interventions to increase depression literacy since most interventions are based on cognitive-behavioral therapy concepts [53]. According to the stage model of Wright et al. (2015), involving patients in the adaptation process of a questionnaire can be classified as a preliminary stage of patient and public involvement (PPI) [54]. By conducting cognitive interviews with patients, we aimed to promote a participatory research approach, which cannot yet be regarded as standard in Germany [55].

### Limitations

The study has some limitations, one of which is due to the online nature of the study. Because the study was online, subjects could search for the correct answers while completing the scale to achieve a better test result. Due to this limitation, we calculated a correlation between the processing time and the total score. Processing time correlated negatively with the total score. This could mean that participants who knew less about depression spent more time thinking about the answers and had a longer processing time. However, it might be that those who knew less would have scored even worse if they had not used the processing time for research. In the future, a time limit should be implemented to prevent this possible bias. Despite the achievement of the minimum sample size, the sample was not representative (young average age, high level of education). Since we conducted the scale on a convenient sample, due to the online nature of the study, younger participants might have had easier access to the study. Furthermore, women are shown to participate in online surveys more often than men [56]. This was also the case in our study, which might have led to higher depression knowledge since women have higher depression knowledge and mental health literacy in general [6, 7, 57, 58]. The high proportion of correct answers could also be traced back to the educational level of the sample, which was above average. In addition, 77% of individuals reported having depression experience, and 43% reported having treatment experience. Overall, 21% of the respondents were undergoing treatment for their own depression at the time they participated in the study. This aligns with the results from insurance fund routine data in Germany [4, 59], but could also have been favorable for a higher depression knowledge. To summarize, the results of this study are not representative of the general population, as the people included in the study were predominantly young, highly educated, and female.

Nevertheless, our results can suggest a preliminary factor structure that should be tested again using a representative sample. Further studies should focus on more inclusive strategies to recruit participants and consider implementing the scale in a paper-based format. Instead of focusing on universities or other educational institutions for recruitment, health care centers, e.g., general practices, community health care centers, or clinics, should be considered. Also, to avoid only attracting people who have already experienced depression, the topic of the research project should not be revealed until the end of the survey. Considering the moderate selectivities of the items, the absence of an analysis of the test–retest reliability, and the limitations regarding the study population, the German D-Lit-R scale [27] still has to undergo further development and evaluation.

## Conclusion

In the present study, we revised the Geman Depression Literacy scale and evaluated the psychometric values and factor structure of it in a convenient sample. The results indicate that the D-Lit-R German scale measures knowledge of 1) symptoms of depression, 2) symptoms of other psychological disorders that have to be distinguished from depression, and 3) the treatment of depression (pharmacological and psychotherapeutic). The scale conveys satisfactory reliability and can be easily applied since it is very time-saving and standardized. Due to several limitations, such as the limited generalizability resulting from our convenient sample, the scale should be conducted again in a more representative population. Also, further revision is needed to construct a scale that can capture all aspects of depression literacy as defined in the literature and can be used for follow-up measures.

### Supplementary Information

**Supplementary Material 1.**

## Data Availability

The datasets used and analyzed during the current study are available from the corresponding author upon reasonable request.
