# Supplementary material for: The revision and factor analytic evaluation of the German version of the depression literacy scale (D-Lit-R German)
Source: BMC Psychol. 2024 Apr 25;12:235. doi: 10.1186/s40359-024-01730-9 (PMC11046899; doi:10.1186/s40359-024-01730-9)
Supplement: Supplementary file 1 — Supplementary Material 1. [file 40359_2024_1730_MOESM1_ESM.zip › D-Lit-R German scale.docx]

| **German Depression Literacy Scale Revised (D-Lit-R German)**  Kreuzen Sie bitte die richtigen Antwortmöglichkeiten an und nutzen Sie die Antwortoption „ich weiß es nicht“ bitte nur, wenn Sie sich absolut unsicher sind. | | | | |
| --- | --- | --- | --- | --- |
|  |  | Wahr | Falsch | Ich weiß es nicht |
| (1) | Menschen mit Depressionen sprechen oft zusammenhanglos. |  |  |  |
| (2) | Menschen mit Depressionen können sich schuldig fühlen, obwohl sie nichts falsch gemacht haben. |  |  |  |
| (3) | Rücksichtsloses und risikofreudiges Verhalten sind übliche Zeichen von Depressionen. |  |  |  |
| (4) | Verlust von Selbstvertrauen und geringes Selbstwertgefühl können Zeichen von Depressionen sein. |  |  |  |
| (5) | Nicht auf die Fugen eines Fußwegs zu treten, kann ein Zeichen von Depressionen sein. |  |  |  |
| (6) | Menschen mit Depressionen hören oft Stimmen, die nicht da sind. |  |  |  |
| (7) | Zu viel oder zu wenig Schlaf kann ein Zeichen von Depressionen sein. |  |  |  |
| (8) | Zu viel essen oder den Appetit verlieren können Zeichen von Depressionen sein. |  |  |  |
| (9) | Depressionen haben keinen Einfluss auf Gedächtnis und Konzentration. |  |  |  |
| (10) | Es kann ein Zeichen von Depressionen sein, wenn man mehrere unterschiedliche Persönlichkeiten hat. |  |  |  |
| (11) | Infolge einer Depression kann es sein, dass Menschen sich langsamer bewegen oder ganz ruhelos sind. |  |  |  |
| (12) | Psycholog:innen können Antidepressiva verschreiben. |  |  |  |
| (13) | Depressionen können mit Veränderungen im Denken und in der Wahrnehmung einhergehen (z.B. Grübeln). |  |  |  |
| (14) | Die meisten Menschen mit Depressionen müssen ins Krankenhaus eingeliefert werden. |  |  |  |
| (15) | Depressionen haben immer mehrere Ursachen. |  |  |  |
| (16) | Die Steigerung positiver Aktivitäten (z.B. Bewegung, Kontakte) sollte ein fester Bestandteil jeder Depressionsbehandlung sein. |  |  |  |
| (17) | Bei Depressionen ist Beratung genauso wirksam wie kognitive Verhaltenstherapie. |  |  |  |
| (18) | Bei leichten bis mittelschweren Depressionen ist Kognitive Verhaltenstherapie die Behandlung erster Wahl. |  |  |  |
| (19) | Menschen mit Depressionen haben negative Gedanken, die sich auch in Form von Suizidgedanken äußern können. |  |  |  |
| (20) | Menschen mit Depressionen sollten aufhören Antidepressiva zu nehmen, sobald sie sich besser fühlen. |  |  |  |
| (21) | Antidepressiva machen süchtig. |  |  |  |
| (22) | Antidepressiva wirken in der Regel sofort. |  |  |  |

Gökce, F., Jais, D. & Pitschel-Walz, G. (2022). *German Depression Literacy Scale Revised (D-Lit-R German).* Institute of General Practice and Health Services Research, TU Munich.
