# Supplementary material for: The revision and factor analytic evaluation of the German version of the depression literacy scale (D-Lit-R German)
Source: BMC Psychol. 2024 Apr 25;12:235. doi: 10.1186/s40359-024-01730-9 (PMC11046899; doi:10.1186/s40359-024-01730-9)
Supplement: Supplementary file 1 — Supplementary Material 1. [file 40359_2024_1730_MOESM1_ESM.zip › D-Lit-R German_English.docx]

| **German Depression Literacy Scale Revised (D-Lit-R German)**  The following statements regarding depression can be true or false. Please tick the right answer option. Only use the answer option "I don't know" if you are absolutely unsure. | | | | |
| --- | --- | --- | --- | --- |
|  |  | True | False | I don´t know |
| (1) | People with depression often speak incoherently. |  |  |  |
| (2) | People with depression can feel guilty even though they have done nothing wrong. |  |  |  |
| (3) | Reckless and risk-taking behaviour are common signs of depression. |  |  |  |
| (4) | Loss of self-confidence and low self-esteem can be signs of depression. |  |  |  |
| (5) | Not stepping on the joints of a footpath can be a sign of depression. |  |  |  |
| (6) | People with depression often hear voices that are not there. |  |  |  |
| (7) | Sleeping too much or too little can be a sign of depression. |  |  |  |
| (8) | Eating too much or losing your appetite can be signs of depression. |  |  |  |
| (9) | Depression does not affect memory and concentration. |  |  |  |
| (10) | Having several different personalities can be a sign of depression. |  |  |  |
| (11) | As a result of depression, people may move more slowly or be completely restless. |  |  |  |
| (12) | Psychologists can prescribe antidepressants. |  |  |  |
| (13) | Depression can be accompanied by changes in thinking and perception (e.g. brooding). |  |  |  |
| (14) | Most people with depression need to be admitted to hospital. |  |  |  |
| (15) | Depression always has several causes. |  |  |  |
| (16) | Increasing positive activities (e.g. exercise, socializing) should be an integral part of any depression treatment. |  |  |  |
| (17) | For depression, counselling is as effective as cognitive behavioural therapy. |  |  |  |
| (18) | For mild to moderate depression, cognitive behavioural therapy is the treatment of choice. |  |  |  |
| (19) | People with depression have negative thoughts, which can also take the form of suicidal thoughts. |  |  |  |
| (20) | People with depression should stop taking antidepressants as soon as they feel better. |  |  |  |
| (21) | Antidepressants are addictive. |  |  |  |
| (22) | Antidepressants usually work immediately. |  |  |  |

Gökce, F., Jais, D. & Pitschel-Walz, G. (2022). *German Depression Literacy Scale Revised (D-Lit-R German).* Institute of General Practice and Health Services Research, TU Munich.
