# Supplementary material for: The revision and factor analytic evaluation of the German version of the depression literacy scale (D-Lit-R German)
Source: BMC Psychol. 2024 Apr 25;12:235. doi: 10.1186/s40359-024-01730-9 (PMC11046899; doi:10.1186/s40359-024-01730-9)
Supplement: Supplementary file 1 — Supplementary Material 1. [file 40359_2024_1730_MOESM1_ESM.zip › Flyer.pdf]

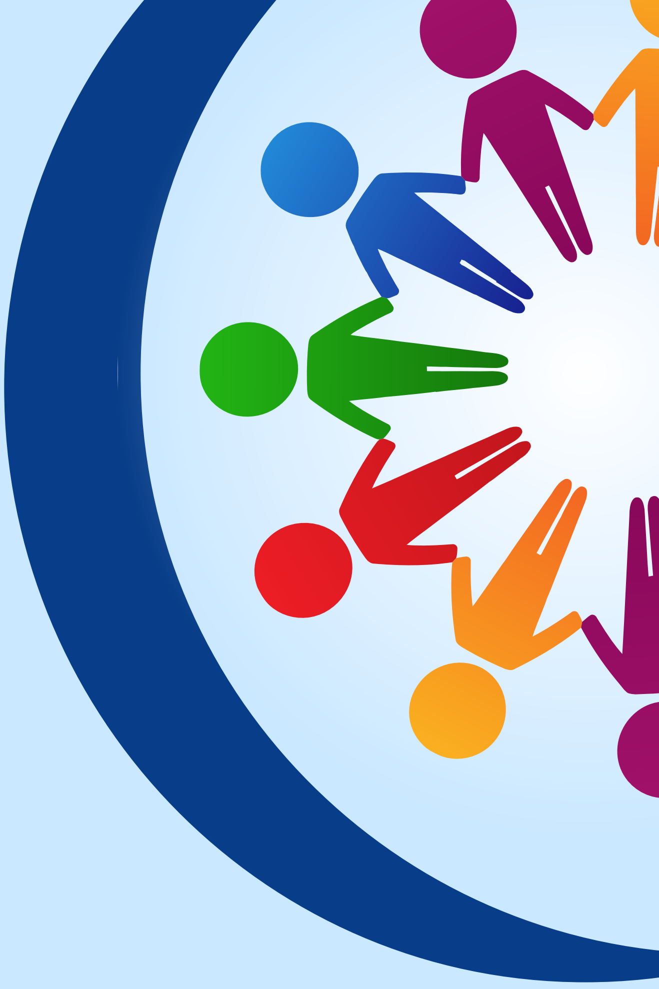

WIR WOLLEN'S WISSEN - SIE AUCH?

Wie gut ist Ihr Wissen über Depressionen?

Finden Sie es heraus und nehmen Sie an unserer **5-10 minütigen Befragung** teil.

WORUM GEHT ES?

Wir möchten die Versorgung von Menschen mit depressiven Erkrankungen gerne verbessern. Mit Ihrer Unterstützung soll dafür ein Wissensfragebogen zur Depression verbessert werden.

ABLAUF:

Füllen Sie den Online-Fragebogen aus und erhalten Sie anschließend Ihren persönlichen Punktescore.

VORAUSSETZUNGEN:

- 18 bis 80 Jahre alt
- **keine** kognitiven &/oder sprachlichen Beeinträchtigungen, die eine Bearbeitung des Fragebogens erschweren
- **keine** Personen mit psychologischem/psychiatrischem Berufshintergrund
- **keine** Psychologiestudierenden

INTERESSIERT?

Scannen Sie bequem den QR-Code und schon sind Sie dabei!

SCAN ME

HERZLICHEN DANK FÜR IHRE TEILNAHME!
DENISE JAIS (B.SC. PSYCHOLOGIE)

Klinikum rechts der Isar

Technische Universität München
