# Supplementary material for: The revision and factor analytic evaluation of the German version of the depression literacy scale (D-Lit-R German)
Source: BMC Psychol. 2024 Apr 25;12:235. doi: 10.1186/s40359-024-01730-9 (PMC11046899; doi:10.1186/s40359-024-01730-9)
Supplement: Supplementary file 1 — Supplementary Material 1. [file 40359_2024_1730_MOESM1_ESM.zip › Reanalysis of the 3-factor model after removing Item 15.docx]

Reanalysis of the 3-factor model after removing Item 15

|  | | | | | |  |
| --- | --- | --- | --- | --- | --- | --- |
| **Table 7** |  | |  | | | |
| Factor loadings on a 3-factor model after removing Item 15 | | | | | | |
|  |  | | Factors | | | |
| Item |  | *h^2^* | 1 | 2 | 3 | |
| (1) | People with depression often speak incoherently. | .13 | **.20^*^** | .11 | .16 | |
| (3) | Reckless and risk-taking behaviour are common signs of depression. | .13 | **.18^*^** | **.20^*^** | .11 | |
| (5) | Not stepping on the joints of a footpath can be a sign of depression. | .56 | **.79^**^** |  |  | |
| (6) | People with depression often hear voices that are not there. | .74 | **.76^**^** | .10 | **.13^*^** | |
| (10) | Having several different personalities can be a sign of depression. | .83 | **.85^**^** |  | **.14^*^** | |
| (14) | Most people with depression need to be admitted to hospital. | .54 | **.54^**^** | **.29^*^** | .10 | |
| (2) | People with depression can feel guilty even though they have done nothing wrong. | .30 | .10 | **.50^*^** |  | |
| (3) | Reckless and risk-taking behaviour are common signs of depression. | .13 | **.18^*^** | **.20^*^** | .11 | |
| (4) | Loss of self-confidence and low self-esteem can be signs of depression. | .34 |  | **.62^**^** |  | |
| (7) | Sleeping too much or too little can be a sign of depression. | .60 |  | **.72^**^** | .15 | |
| (8) | Eating too much or losing your appetite can be signs of depression. | .79 |  | **.88^**^** | .11 | |
| (9) | Depression does not affect memory and concentration. | .26 |  | **.42^**^** | **.19*** | |
| (11) | As a result of depression, people may move more slowly or be completely restless. | .23 |  | **.45^**^** | **.20^*^** | |
| (13) | Depression can be accompanied by changes in thinking and perception (e.g. brooding). | .33 | .19 | **.49^**^** |  | |
| (16) | Increasing positive activities (e.g. exercise, socializing) should be an integral part of any depression treatment. | .12 | .16 | **.29^*^** |  | |
| (19) | People with depression have negative thoughts, which can also take the form of suicidal thoughts. | .74 | **.30^*^** | **.76^**^** |  | |
|  |  |  |  |  |  | |
| (12) | Psychologists can prescribe antidepressants. | .44 | **.23^*^** | **.27^**^** | **.39^**^** | |
| (17) | For depression, counselling is as effective as cognitive behavioural therapy. | .25 | **.18^*^** |  | **.39^**^** | |
| (18) | For mild to moderate depression, cognitive behavioural therapy is the treatment of choice. | .09 |  | .12 | **.23^*^** | |
| (20) | People with depression should stop taking antidepressants as soon as they feel better. | .63 | .11 |  | **.76^**^** | |
| (21) | Antidepressants are addictive. | .71 | **.20^*^** |  | **.75^**^** | |
| (22) | Antidepressants usually work immediately. | .66 |  | **.16^*^** | **.78^**^** | |
| *Note. **p <* .001, **p* < .05. *λ* = loadings, *h^2^* = commonalities. For clarity, factor loading < .10 are left blank. | | | | | | |
